# Bacterial Outer Membrane Vesicles in Potentiating Cancer Vaccines: Progress and Prospects

**DOI:** 10.1002/advs.202521684

**Published:** 2026-02-19

**Authors:** Jiabeini Zhang, Tianyu Shao, Fengjiang Qu, Caiyun Bai, Sijie Li, Pin Gao, Wenna Li, Keman Cheng, Xiao Zhao, Zhimin Fan

**Affiliations:** ^1^ Department of Breast Surgery, General Surgery Center The First Hospital of Jilin University Changchun Jilin China; ^2^ CAS Key Laboratory for Biomedical Effects of Nanomaterials and Nanosafety & CAS Center for Excellence in Nanoscience National Center for Nanoscience and Technology of China Beijing China; ^3^ Guang' anmen Hospital China Academy of Chinese Medical Sciences Beijing China; ^4^ Department of Emergency Surgery The First Hospital of Jilin University Changchun Jilin China

**Keywords:** bacterial engineering, cancer immunotherapy, cancer vaccines, hybrid membrane vesicles, outer membrane vesicles, surface modification

## Abstract

Cancer immunotherapy is increasingly moving toward personalized, precision‐based strategies, with cancer vaccines emerging as a promising approach to reshape treatment. However, despite their potential, current tumor vaccines often yield limited clinical responses and subpar immunogenicity, underscoring the urgent need for innovative delivery systems to enhance immune activation. Bacterial outer membrane vesicles (OMVs), which possess natural immunomodulatory properties and impressive engineering flexibility, have attracted attention as versatile platforms for vaccine development and bioengineering applications. This review thoroughly summarizes recent advances in using OMVs to enhance the effectiveness of cancer vaccines. First, we explain the key biological features of OMVs that support their immunotherapeutic potential. Next, we carefully analyze the primary mechanisms by which OMVs enhance immune responses, as well as cutting‐edge engineering strategies to improve their safety, immunogenicity, and specificity. Additionally, we discuss the significant challenges that hinder the clinical use of OMV‐based cancer vaccines and provide a comprehensive review of current progress and future outlooks. Looking forward, combining artificial intelligence, tumor microenvironment profiling, and neoantigen discovery is expected to drive the development of next‐generation, personalized OMV‐based immunotherapies. Overall, OMVs stand out as a transformative platform capable of overcoming major obstacles in cancer vaccine development and pushing forward future cancer immunotherapy.

## Graphical abstract

Bacterial outer membrane vesicles (OMVs) have emerged as versatile platforms for cancer vaccine development owing to their intrinsic immunostimulatory properties and high engineering flexibility. This review summarizes OMV biology, immune mechanisms, and engineering strategies that enhance vaccine efficacy, discusses key translational challenges, and highlights future opportunities for personalized cancer immunotherapy.

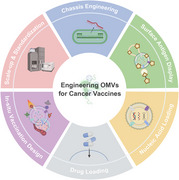

## 1 Introduction

Cancer remains one of the leading global health challenges, with incidence and mortality continuing to rise worldwide. Despite advances in surgery, chemotherapy, radiotherapy, and targeted therapies, treatment outcomes for metastatic, immune‐evasive, and therapy‐resistant tumors remain unsatisfactory, often accompanied by recurrence and systemic toxicity [1]. In recent years, cancer immunotherapy—particularly immune checkpoint inhibitors (ICIs) and adoptive cell therapies‐ has substantially improved clinical outcomes in several malignancies, including melanoma and non‐small cell lung cancer [2, 3]. However, heterogeneous therapeutic responses, limited patient coverage, high costs, and treatment‐associated toxicities continue to restrict their broader application, highlighting the need for safer, more durable, and broadly applicable immunotherapeutic strategies [4, 5].

Tumor vaccines represent a central pillar of cancer immunotherapy by aiming to establish long‐term immune surveillance through antigen‐specific cytotoxic T lymphocyte activation. Nevertheless, current vaccine platforms, including peptide‐, mRNA‐, and dendritic cell–based vaccines, have achieved only limited clinical success due to insufficient immunogenicity, inefficient antigen delivery, and suppression by the tumor immune microenvironment [6, 7, 8, 9]. Bacterial outer membrane vesicles (OMVs) are nanoscale vesicles released by Gram‐negative bacteria and were first identified in 1965 through observations of lipopolysaccharide (LPS) release without cell lysis in *Escherichia coli* (*E. coli)* [10]. Typically 20–250 nm in size, OMVs are enriched in pathogen‐associated molecular patterns (PAMPs), which are sensed by dendritic cells (DCs) via pattern recognition receptors (PRRs), including toll‐like receptors (TLRs) and NOD‐like receptors (NLRs), thereby promoting antigen presentation and activating innate and adaptive immune responses [11]. Beyond their intrinsic immunostimulatory properties, OMVs exhibit remarkable structural and engineering versatility. Their membrane architecture and luminal space can be genetically or chemically modified to enable programmable antigen display, modular functionalization, and co‐delivery of immunoregulatory molecules, including through systems such as SpyTag/SpyCatcher. Moreover, OMVs have been shown to induce trained immunity by reprogramming the metabolic and transcriptional states of myeloid precursor cells, thereby enhancing antigen presentation, reshaping the phenotypes of tumor‐associated macrophages (TAMs), and alleviating immunosuppressive features of the tumor microenvironment (TME) [12, 13, 14].

Owing to their distinct immunological functions and structural versatility, OMVs are emerging as a versatile and engineerable platform to address key challenges in tumor vaccine development. They not only provoke strong immune responses but can also be engineered for personalized, targeted vaccine designs. Increasing research focus has been placed on combining OMVs with advanced technologies, such as artificial intelligence (AI), TME profiling, and neoantigen prediction, which collectively guide cancer vaccines toward intelligent, precision‐based therapies. Thus, a comprehensive overview of the biological basis, immune mechanisms, engineering strategies, and translational potential of OMVs will offer valuable theoretical and technical insights for the development of next‐generation tumor vaccines, thereby expanding the scope of cancer immunotherapy research.

## 2 Biological Characteristics of OMVs Related to Tumor Vaccines

OMVs are nanoscale membranous vesicles released by Gram‐negative bacteria during normal growth or under stress conditions [15]. They play vital roles in bacteria–host interactions, infection dissemination, and immune evasion, and in recent years, have been extensively studied for applications in tumor vaccine development and nanodelivery systems [11, 16].

### 2.1 Composition and Immunogenic Basis

OMVs originate from the outward budding of the bacterial outer membrane and periplasmic space, forming asymmetric bilayer vesicles enriched in immunologically active components, including LPS, lipooligosaccharides (LOS), lipoproteins, outer membrane proteins (e.g., OmpA, PorB), peptidoglycan fragments, bacterial nucleic acids, and glycosylated antigens—collectively functioning as PAMPs [15, 17]. These components enable OMVs to engage multiple pattern recognition receptors (PRRs) on host immune cells, thereby initiating robust innate immune activation.

Among these components, LPS and LOS are key determinants of OMV immunogenicity, with lipid A structure critically influencing the balance between immune activation and endotoxicity. Through TLR2/4‐mediated recognition, lipoproteins and outer membrane proteins activate MyD88–NF‐κB/MAPK signaling pathways, leading to proinflammatory cytokine production. In parallel, peptidoglycan fragments and bacterial nucleic acids stimulate cytosolic and endosomal sensors, including NOD1/2, NLRP3, and TLR7/8/9, thereby coordinating membrane‐ and cytosol‐derived immune signaling. Table 1 summarizes the major OMV components, their corresponding PRRs, and immune functions, highlighting the structure–function relationships underlying OMV immunogenicity.

**TABLE 1:** Immunological Mechanisms and Functional Roles of Major OMV Components.

PAMPs	Primary sources	PRRs	Primary responsive cell types	Principal immunological functions and mechanisms	References
LPS	Outer membrane of Gram‐negative bacteria	TLR4/MD‐2/CD14 (membrane/endosome); Caspase‐11 (cytosol)	DCs, monocytes/macrophages, endothelial cells	LPS is a crucial immunoactive component of OMVs; the structure of lipid A affects immunogenicity and toxicity. Recognition via TLR4–MyD88/TRIF activates NF‐κB and MAPK pathways, triggering the release of proinflammatory cytokines and promoting DC maturation and antigen presentation.	[152, 153, 154, 155]
LOS	Outer membrane of Gram‐negative bacteria	TLR4/MD‐2, TLR2/6 (membrane); Caspase‐11 (cytosol)	DCs, macrophages, epithelial cells	LOS lacking O‐antigen chains generally shows lower toxicity. Pathogenic LOS activates TLR4–MyD88/TRIF and Caspase‐11, leading to IL‐1β and TNF‐α production; structural changes can lessen endotoxin activity while maintaining immune activation.	[156, 157]
Lipoproteins	Outer‐membrane–anchored proteins	TLR1/2 or TLR2/6 (membrane)	DCs, macrophages, neutrophils	Major immunoactive components, acylation state, and LES sequence co‐regulate immune recognition and vesiculation. The TLR2 signaling pathway promotes the release of proinflammatory cytokines and enhances antigen presentation.	[20, 158, 159, 160, 161]
Outer membrane proteins (e.g., OmpA, PorB)	Outer membrane porins/channel proteins	TLR2, TLR4 Outer membrane porins and channel proteins(membrane)	Epithelial cells, DCs, macrophages, B cells	Serve as structural antigens that improve uptake and cross‐presentation; PorB induces DC maturation via TLR2, and OmpA activates macrophages and increases cytokine secretion, linking innate and adaptive immunity.	[158, 159, 162]
Peptidoglycan	Cell‐wall scaffold component	NOD1/NOD2 (cytosol); TLR2 (membrane, partial consensus); NLRP3 (cytosol)	Epithelial cells, DCs, macrophages, neutrophils	Delivered into the cytosol of non‐phagocytic cells to enable NOD1 recognition of iE‐DAP motifs, activating NF‐κB and MAPK pathways, and inducing IL‐8; also interacts with the NLRP3 inflammasome to promote IL‐1β maturation and strengthen innate immunity.	[158, 159, 163, 164]
Bacterial nucleic acids	Genomic DNA, rRNA, ssRNA	TLR9 (DNA, endosome); TLR7/8 (RNA, endosome)	plasmacytoid DCs, macrophages, B cells	CpG‐DNA or ssRNA activates MyD88–IRF7/NF‐κB signaling, leading to the production of IFN‐I and IL‐6, and enhances B‐cell humoral responses and cross‐presentation.	[158, 159]
Flagellin	Bacterial flagella	TLR5 (membrane)	Epithelial cells, DCs, macrophages	Through TLR5, it activates the MyD88–NF‐κB/MAPK pathway to induce TNF‐α, IL‐6, and other proinflammatory cytokines, thereby promoting DC maturation and chemokine expression.	[158, 159]
Glycoconjugate antigens/polysaccharides	O‐antigen polysaccharide chains of LPS, outer‐membrane glycoproteins, and EPS^a^; tumor‐associated glycans^b^ can be added via engineering	TLR4 (membrane); C‐type lectin receptors (CLRs: DC‐SIGN, Dectin‐1, Mincle)	DCs, B cells, macrophages	Glycosylation affects OMV–host receptor interactions: O‐antigen extension can shield lipid A and reduce TLR4‐driven inflammation; specific glyco‐epitopes (e.g., Lewis antigens) engage DC‐SIGN/Mincle to steer Th1/Th2 responses and boost B‐cell activation.	[11, 86, 162, 165]

^a^ EPS: Extracellular polysaccharides;

^b^ Tumor‐associated glycans such as Tn, sTn, and Lewis antigens.

Collectively, these immune‐active cargos promote DC maturation and enhance antigen uptake, processing, and cross‐presentation, effectively linking innate and adaptive immunity [18]. OMVs also modulate macrophage function by enhancing phagocytosis and promoting polarization toward a proinflammatory M1 phenotype, characterized by increased secretion of cytokines such as IL‐12 and TNF‐α, reduced immunosuppressive signaling within the tumor TME, and enhanced infiltration of cytotoxic T lymphocytes (CTLs) [19, 20]. Beyond DCs and macrophages, OMVs can activate epithelial cells, B cells, and neutrophils through specific surface components and nucleic acid cargos, collectively shaping a multilayered immune response. Importantly, subtle structural variations—such as lipid A acylation patterns, lipoprotein modifications, and O‐antigen chain length—can markedly influence PRR engagement and immune response bias, providing opportunities for precise immunomodulatory tuning.

The immunogenicity of OMVs can be further tailored through rational engineering strategies. Genetic modification of bacterial strains or targeted alteration of lipid A biosynthesis pathways can substantially reduce endotoxin activity while preserving TLR4 agonism, achieving a favorable balance between safety and immune activation [21, 22, 23, 24]. In parallel, materials‐based approaches—such as calcium phosphate (CaP) shell coating—can mitigate systemic toxicity, enhance circulation stability, and introduce stimuli‐responsive properties, while simultaneously promoting macrophage reprogramming toward an antitumor phenotype and enabling multifunctional therapeutic integration [25].

Overall, these biological characteristics and engineering opportunities establish OMVs as highly adaptable and controllable immune platforms, providing a mechanistic foundation for their application in tumor vaccine development and subsequent engineering strategies discussed below.

### 2.2 Interaction With the Host Immune System

Once introduced into the host, OMVs are efficiently internalized by antigen‐presenting cells (APCs), including DCs, macrophages, and monocytes, enabling the processing and presentation of OMV‐associated antigens on major histocompatibility complex (MHC) molecules and subsequent activation of CD4^+^ and CD8^+^ T cells [12, 26, 27, 28, 29]. Importantly, beyond antigen delivery, OMVs provide potent innate immune stimulation that establishes a permissive activation context for effective antigen presentation, thereby facilitating immune recognition of weak tumor‐associated antigens [30, 31]. In addition, OMVs can induce trained immunity in myeloid cells through epigenetic reprogramming, leading to enhanced responsiveness upon secondary stimulation or exposure to heterologous antigens, which may contribute to more rapid tumor control following relapse [14, 32, 33].

In summary, the unique composition and immunogenic properties of OMVs, together with their multifaceted interactions with the host immune system, underpin their advantages as tumor vaccine platforms. Nevertheless, how OMVs coordinate innate and adaptive immunity, reshape the TME, and induce immunogenic cell death (ICD) remains incompletely understood. These mechanistic aspects are discussed in the following section.

## 3 Mechanistic Insights Into OMV‐Enhanced Tumor Vaccine Effectiveness

### 3.1 Activation of Innate Immunity and Trained Immunity

PAMPs enriched in OMVs are recognized by host PRRs, triggering downstream inflammatory signaling pathways and recruiting various immune cell types. This process not only initiates acute innate immune responses but also establishes long‐term trained immunity through epigenetic and metabolic reprogramming, thereby connecting innate and adaptive immunity.

#### 3.1.1 Neutrophils as Rapid Responders to Inflammation

Neutrophils are among the earliest responders during OMV‐induced innate immune activation. Upon OMV stimulation, vascular endothelial cells rapidly secrete chemokines such as IL‐8 and CXCL1 via the TLR4–NF‐κB signaling axis, establishing a chemotactic gradient that drives efficient neutrophil recruitment at early inflammatory stages [34].

The magnitude and duration of neutrophil responses depend on the bacterial origin of OMVs. *E. coli*–derived OMVs induce acute neutrophilic inflammation primarily through the TLR4–IL‐8 axis, whereas OMVs from pulmonary commensal bacteria sustain CXCL1 expression via the TLR–MyD88–IL‐17B pathway, promoting prolonged inflammatory signaling [35]. In contrast, *Pseudomonas aeruginosa* OMVs carrying small RNAs (sRNA52320) suppress IL‐8 production in host cells, thereby limiting neutrophil recruitment and reflecting a bacterial immune‐evasion strategy [36, 37].

Activated neutrophils then release TNF‐α and IL‐1β and form neutrophil extracellular traps (NETs), web‐like DNA–protein structures that capture pathogens or tumor antigens. This amplifies local inflammation and increases antigen accessibility, aiding in antigen uptake and presentation by macrophages and DCs [38, 39].

#### 3.1.2 Macrophages as Amplifiers of Immune Responses

Under OMV stimulation, macrophages phagocytose vesicles and activate inflammasome pathways, releasing IL‐1β and IL‐18 while reprogramming from an immunosuppressive M2 phenotype to a proinflammatory M1 phenotype [40, 41, 42, 43, 44]. This transition improves antigen processing and presentation, diminishes immunosuppressive signaling within the TME, and creates conditions that favor T‐cell infiltration and activation. Additionally, M1 macrophages secrete CXCL9/10 to promote DC migration to lymph nodes and, through vesicular communication or cell–cell contact, enhance DC antigen‐processing efficiency, thereby facilitating adaptive immune priming [45, 46, 47].

#### 3.1.3 OMV‐Induced Trained Immunity

Beyond triggering acute inflammation, OMVs can establish long‐term trained immunity within the monocyte–macrophage lineage, giving innate immune cells memory‐like responses. PAMPs in OMVs activate the NLRP3–Caspase‐1–IL‐1β inflammasome pathway in bone marrow monocyte precursors and stimulate the mTOR–HIF‐1α metabolic axis, leading to IL‐1β release and metabolic reprogramming [14, 48]. These metabolic cues induce stable epigenetic remodeling, characterized by enrichment of histone marks such as H3K4me3, which reshapes the transcriptional landscape of hematopoietic progenitors and generates proinflammatory trained monocytes. Upon peripheral differentiation, these cells adopt an M1‐like phenotype with sustained secretion of IL‐1β, TNF‐α, and IL‐6, as well as CXCL9/10, thereby reinforcing DC maturation, antigen presentation, and T‐cell activation. Distinct from transient immune activation, this hierarchical “metabolic–epigenetic–functional” reprogramming enables rapid and amplified secondary immune responses upon antigen re‐exposure, substantially enhancing the immunogenicity and durability of OMV‐based tumor vaccines. Leveraging this principle, an engineered OMV‐SIRPα@CaP/GM‐CSF system has been developed, in which granulocyte–macrophage colony‐stimulating factor (GM‐CSF) promotes bone marrow progenitor mobilization and M1 polarization, while signal regulatory protein‐α (SIRPα)‐Fc disrupts the CD47–SIRPα immune checkpoint, synergistically augmenting macrophage phagocytosis and sustaining DC‐ and T cell–mediated antitumor immunity [13].

#### 3.1.4 DCs as Central Orchestrators

OMV stimulation prompts DCs to release IL‐12 and TNF‐α while increasing MHC‐II and costimulatory molecules (CD80, CD86), significantly enhancing antigen presentation. Mature DCs then activate CD4^+^ helper T cells and CTLs, with their maturity level directly affecting the strength and specificity of adaptive immune responses [49, 50, 51, 52, 53].

**FIGURE 1:**
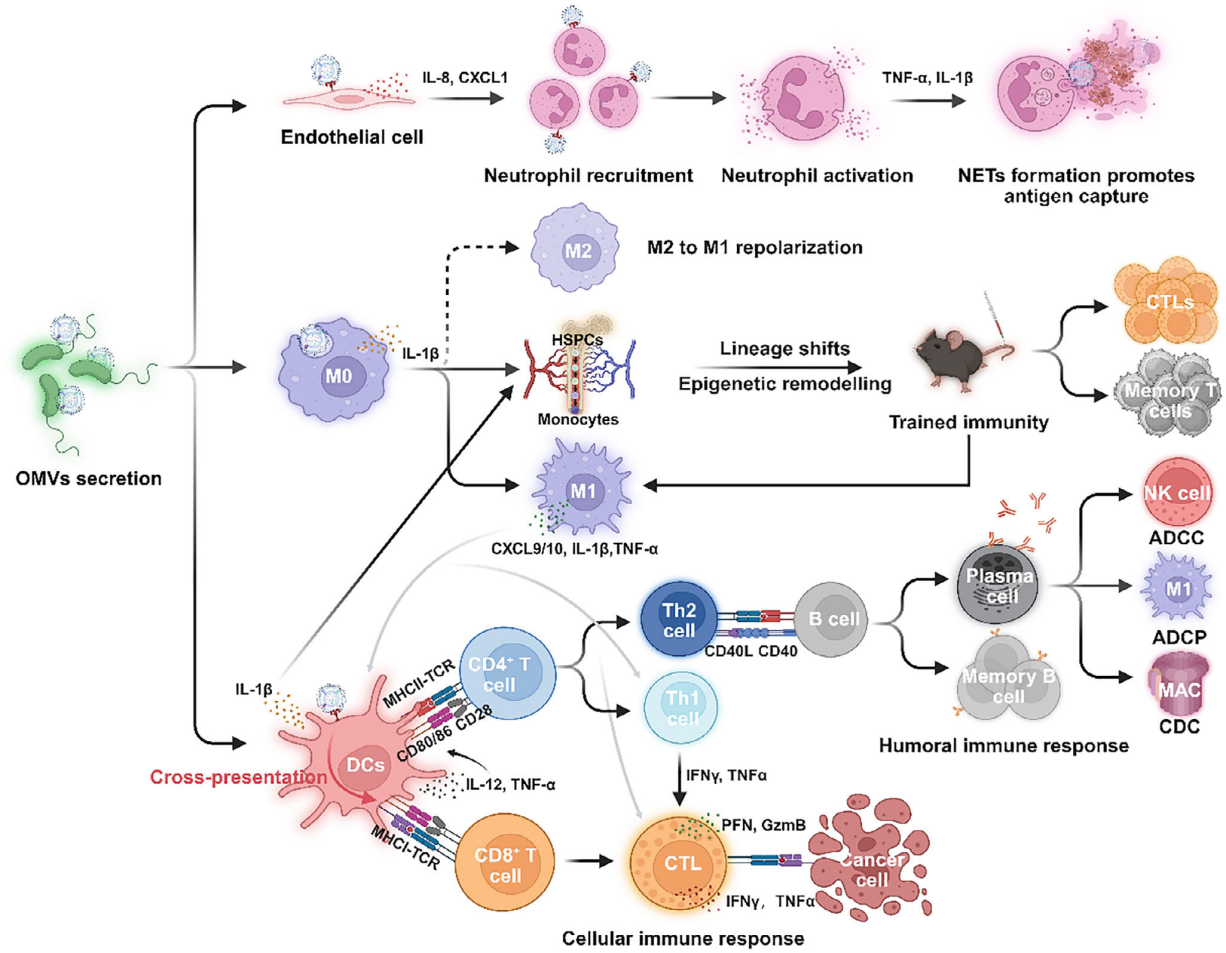
Schematic illustration of the immunological mechanisms by which OMVs induce antitumor immune activation. The surface PAMPs of OMVs are recognized by host PRRs, triggering endothelial cells to secrete IL‐8 and CXCL1 that recruit neutrophils. Activated neutrophils release TNF‐α and IL‐1β and form NETs that capture and expose antigens. Subsequently, macrophages engulf OMVs and activate the inflammasome pathway (NLRP3–Caspase‐1–IL‐1β), releasing IL‐1β and IL‐18 and polarizing from the M2 to proinflammatory M1 state. Concurrently, OMVs reprogram bone marrow–derived hematopoietic stem and progenitor cells (HSPCs) via the mTOR–HIF‐1α metabolic axis, inducing metabolic and epigenetic remodeling that supports trained immunity. This process establishes memory‐like innate immune responses, enabling monocyte–macrophage lineages to respond more robustly upon secondary stimulation. The development of trained immunity drives macrophage M1 polarization, and CXCL9/10 secretion by these cells promotes DC maturation and antigen presentation. Mature DCs activate both MHC‐I and MHC‐II pathways, facilitating cross‐presentation of exogenous antigens and the activation of CD4^+^ and CD8^+^ T cells. Overall, OMVs integrate innate immune activation, trained immunity formation, and adaptive immune amplification to construct a systemic immune network that markedly enhances the efficacy of tumor vaccines.

In summary, by rapidly activating neutrophils, amplifying responses via macrophages, and presenting antigens via DCs, OMVs effectively stimulate innate immunity and lay a strong foundation for adaptive immune activation. Figure 1 shows the multi‐level coordination of OMV‐induced innate immune activation, trained immunity development, and adaptive immune enhancement at both cellular and molecular levels.

### 3.2 Antigen Presentation and Adaptive Immune Response

As nanoscale vesicles, OMVs possess inherent advantages in lymphatic drainage, allowing efficient entry into secondary lymphoid organs where they are readily captured by DCs, thereby promoting antigen presentation and the establishment of adaptive immune responses. In this process, DCs act as the central orchestrators: on one hand, mature DCs present antigens to CD4^+^ T helper cells via MHC‐II molecules, enhancing Th1/Th2 immune responses; on the other hand, they mediate cross‐presentation by loading exogenous antigens onto MHC‐I molecules, thereby activating CTLs and inducing potent cytotoxic effects. This mechanism breaks the traditional limitation that “exogenous antigens rely solely on the MHC‐II pathway” and is regarded as a key process through which OMVs potentiate tumor vaccine efficacy [10, 54, 55, 56, 57].

Furthermore, with advances in bioengineering, OMVs can be genetically modified or engineered to display surface antigens and carry tumor‐associated antigens. For instance, the recently developed “Plug‐and‐Display” technology enables rapid, modular presentation of multiple tumor antigens on OMV surfaces, thereby eliciting polyvalent immune responses and providing a feasible pathway for the development of personalized tumor vaccines [12, 15, 58]. Therefore, OMVs integrate intrinsic adjuvant effects with efficient antigen‐delivery capabilities, thereby markedly amplifying both cellular and humoral immune responses in a tumor‐specific manner.

### 3.3 Remodeling the TME and Inducing ICD

Following the development of adaptive immune responses, the immunosuppressive environment within the TME remains a significant obstacle to the effective delivery of tumor vaccines. To address this challenge, OMVs not only act as antigen‐delivery platforms but also actively alter the tumor immune environment through multiple mechanisms, thereby sustaining the immune response. Specifically, this section examines three main aspects of OMV‐mediated modulation: reduction of immunosuppression and cellular reprogramming; induction of ICD; and physicochemical signal–driven synergistic enhancement.

#### 3.3.1 Relief of Immunosuppression and Cellular Reprogramming

OMVs can reverse immunosuppressive conditions within the TME by modulating the phenotypes and signaling networks of tumor‐infiltrating immune cells. Studies have demonstrated that OMVs promote the polarization of TAMs from the immunosuppressive M2 phenotype to the proinflammatory M1 phenotype, while simultaneously attenuating the inhibitory functions of myeloid‐derived suppressor cells and regulatory T cells, thereby restoring local immune activity within the tumor milieu [59]. In parallel, engineered OMVs can induce lysosome‐dependent degradation of PD‐L1, effectively overcoming immune checkpoint resistance and significantly enhancing cytotoxic T cell activation [60, 61]. Thus, OMVs play a central role in the “immunosuppression relief–immune reeducation” process, establishing a favorable foundation for subsequent adaptive immune responses.

#### 3.3.2 Induction of ICD

Beyond immune regulation, OMVs can directly induce ICD, a form of regulated cell death characterized by the release of damage‐associated molecular patterns (DAMPs) that promote antigen presentation and adaptive immune activation, thereby increasing antigen exposure and helping establish durable immune memory. It has been reported that OMVs activate the caspase‐11–gasdermin D pathway to trigger pyroptosis and the release of DAMPs such as calreticulin (CRT), ATP, and high‐mobility group box 1 (HMGB1), which promote DC maturation and CD8^+^ T cell infiltration [62]. Another type of pyroptosis‐inducing OMVs can simultaneously trigger pyroptosis and ICD at tumor sites, leading to sustained antigen release and extended immune memory [63]. By linking the processes of cell death and antigen presentation, OMVs effectively enhance both antigen availability and immune response amplification.

#### 3.3.3 Synergistic Enhancement Through Physicochemical Signals

The integration of OMVs with external physicochemical stimuli further expands their immunomodulatory range. For example, Hf–polyphenol–modified OMVs introduce the high‐Z element hafnium to enhance local energy deposition under radiotherapy and catalyze the decomposition of H_2_O_2_ to alleviate tumor hypoxia, thereby markedly amplifying radiotherapy‐induced ICD signaling [64]. Similarly, metal–polyphenol cage–structured OMVs produce reactive oxygen species (ROS) through chemodynamic therapy (CDT), causing oxidative stress and releasing antigens to promote synergistic immune activation [65]. Additionally, thermosensitive hydrogel–OMV systems allow sustained antigen release and support memory T cell maintenance under photothermal stimulation [66]. Overall, these studies show that OMVs can interact with external physical or chemical cues to amplify immune responses, thereby enhancing their potential as next‐generation tumor vaccine platforms.

In summary, OMVs establish a multilayered, synergistically enhanced immune network by rapidly activating innate immunity, supporting effective antigen presentation, and driving TME reprogramming and ICD induction. This process creates an integrated cascade involving immune activation, antigen presentation, and effector response across molecular, cellular, and tissue levels, leading to a comprehensive boost in tumor vaccine responses. Due to their inherent adjuvanticity, precise antigen‐delivery capacity, and ability to regulate the microenvironment, OMVs are emerging as programmable platforms for immune modulation, providing a strong theoretical and technological basis for future engineering and clinical applications.

## 4 Engineering Strategies for OMV‐Based Tumor Vaccines

In therapeutic cancer vaccination, efficacy is constrained by several fundamental challenges, including insufficient tumor antigen immunogenicity, inefficient antigen presentation and cross‐presentation, a highly immunosuppressive TME, and the difficulty of establishing durable antitumor immune memory. These barriers are particularly prominent in the context of therapeutic cancer vaccines, which must overcome pre‐existing immune tolerance and active immunosuppression within established tumors.

Conventional vaccine platforms often fail to integrate antigen delivery, innate immune activation, and immunomodulation within a single system, thereby limiting the magnitude and durability of antitumor immune responses. In this context, OMVs offer a naturally immunogenic and highly engineerable platform for therapeutic tumor vaccination, owing to their intrinsic PAMPs, nanoscale architecture, and bacterial membrane origin, which together enable efficient antigen delivery and potent innate immune activation. Through rational engineering, OMVs can be further optimized to balance safety and immunogenicity while integrating programmable antigen presentation, immunoregulatory signaling, and TME modulation, thereby enabling coordinated immune activation, amplification, and memory formation.

Accordingly, OMV engineering has evolved into a systematic design framework for therapeutic cancer vaccines, encompassing genetic chassis optimization, antigen programming, cargo loading, in situ vaccination strategies, and scalable manufacturing. As detailed below, these strategies collectively transform OMVs into multifunctional platforms capable of overcoming key limitations of therapeutic cancer vaccines and inducing durable systemic antitumor immunity.

### 4.1 Genetic Engineering of Parental Strains

As discussed earlier, OMVs are enriched with immunostimulatory molecules that promote DC maturation and activate CTLs, thereby eliciting systemic antitumor immune responses. For example, OMVs derived from the intestinal commensal *Parabacteroides distasonis* significantly upregulate CXCL10 expression and increase CD8^+^ T cell infiltration, effectively remodeling the TME [67]. Similarly, *E. coli*–derived OMVs can induce apoptosis and S‐phase arrest in neuroblastoma cells while activating innate and adaptive immune responses. [68] These findings demonstrate that natural OMVs possess inherent immunostimulatory potential. However, their high LPS toxicity, complex proteomic composition, and batch‐to‐batch variability lead to unpredictable immune effects and make standardization difficult. As a result, the research focus has shifted from “utilizing natural immunostimulation” to “constructing controllable chassis strains,” aiming to balance safety and immunogenicity through rational genetic engineering of parent bacteria.

In this paradigm shift, genetic attenuation and proteomic minimization have become breakthroughs. Deleting the *msbB* gene decreases the acylation level of lipid A, which reduces endotoxin activity by about 80%, while still maintaining TLR4 signaling and cytokine secretion [69]. At the same time, systematically removing roughly 60 nonessential outer membrane proteins results in OMVs with better uniformity and higher yield. This approach of “proteomic dimensionality reduction” not only reduces immunological noise but also enhances the stability and predictability of immune responses [70, 71]. Overall, these efforts have turned OMVs from random immunostimulatory vesicles into safe, standardized immune tools, establishing a foundation for future modular functionalization.

**FIGURE 2:**
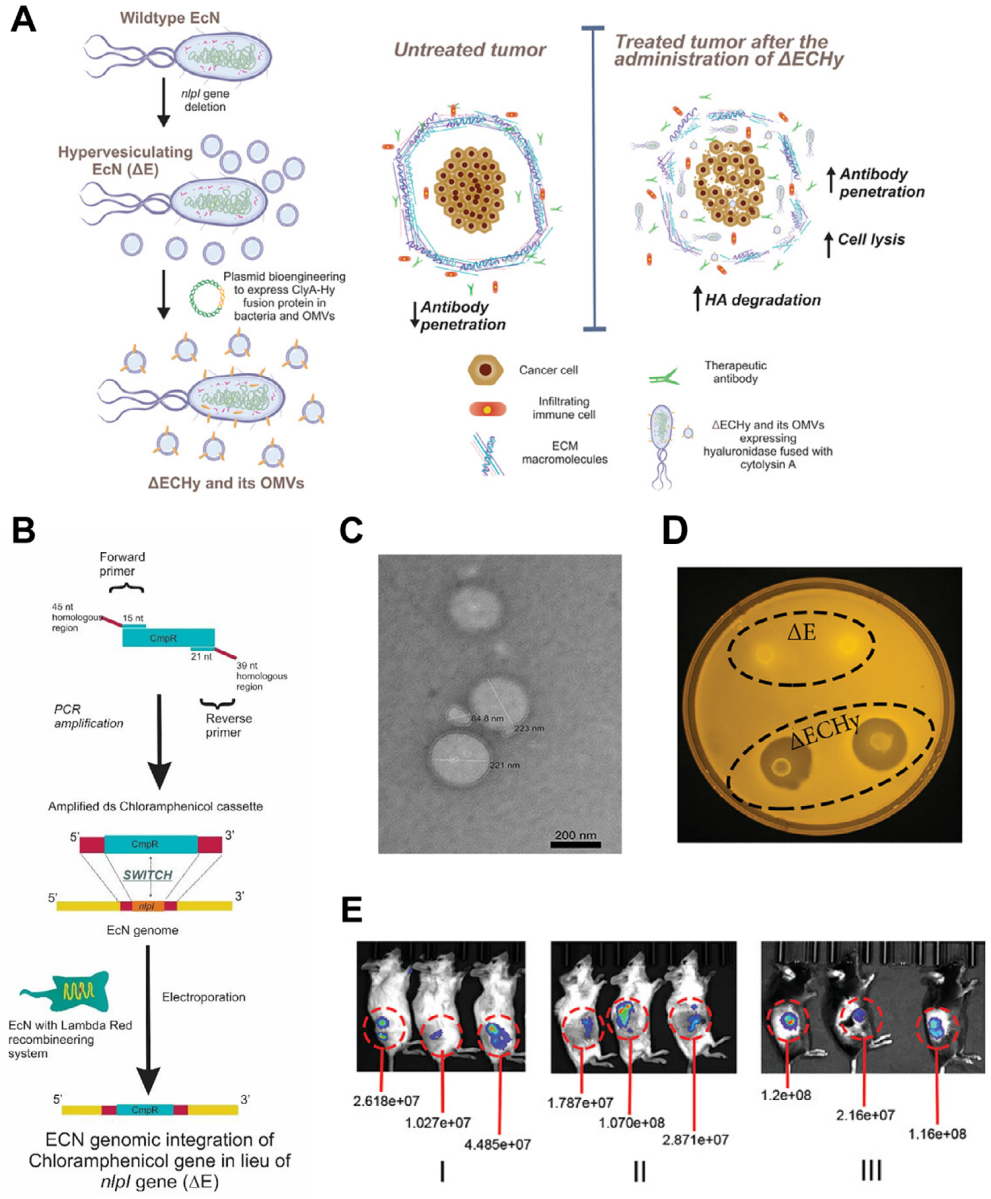
(A) Schematic representing the engineering of *Escherichia coli* Nissle 1917 (EcN) into the ΔECHy strain and a graphical illustration of tumor targeting following systemic administration. Bacteria and their OMVs confined with recombinant hyaluronidase (Hy) are schematically illustrated to be distributed within the tumor matrix, with recombinant Hy targeting HA. (B) Schematic illustration of *nlpI* gene deletion in EcN using λ‐Red homologous recombination to generate the ΔE strain. (C) Representative TEM images of the ΔE strain and the isolated OMVs, showing nanosized vesicles. (D) Qualitative analysis of Hy activity using the HA agarose plate assay. Zones of degradation were observed around the ΔECHy bacteria, whereas ΔE showed no activity. E) Bioluminescence imaging showing tumor accumulation of engineered ΔECHy bacteria at 24 h after intravenous administration. Adapted with permission [72]. Copyright 2021, Wiley‐VCH GmbH.

Importantly, safety optimization of the bacterial chassis represents only the first step in OMV engineering. For example, attaching the extracellular domain of PD‐1 to low‐toxicity OMV surfaces allows simultaneous blockade of the PD‐1–PD‐L1 axis while maintaining the OMV's inherent immunogenicity, thereby creating a synergistic effect with immune checkpoint therapy [69]. Another study showed that deleting the *nlpI* gene significantly enhances OMV biogenesis, enabling the development of a hypervesiculating strain loaded with hyaluronidase. This system facilitates hyaluronic acid (HA) degradation within the tumor extracellular matrix, thereby supporting improved intratumoral distribution of therapeutic agents (Figure 2) [72]. Collectively, these studies demonstrate that rational genetic remodeling not only improves safety and production efficiency but also allows for programmable immune functions. Overall, genetic engineering of parental strains is a critical first step in transforming OMVs from “natural immunostimulatory entities” into purposefully designed immune carriers, providing a solid foundation for subsequent strategies such as antigen display and functional module loading.

**FIGURE 3:**
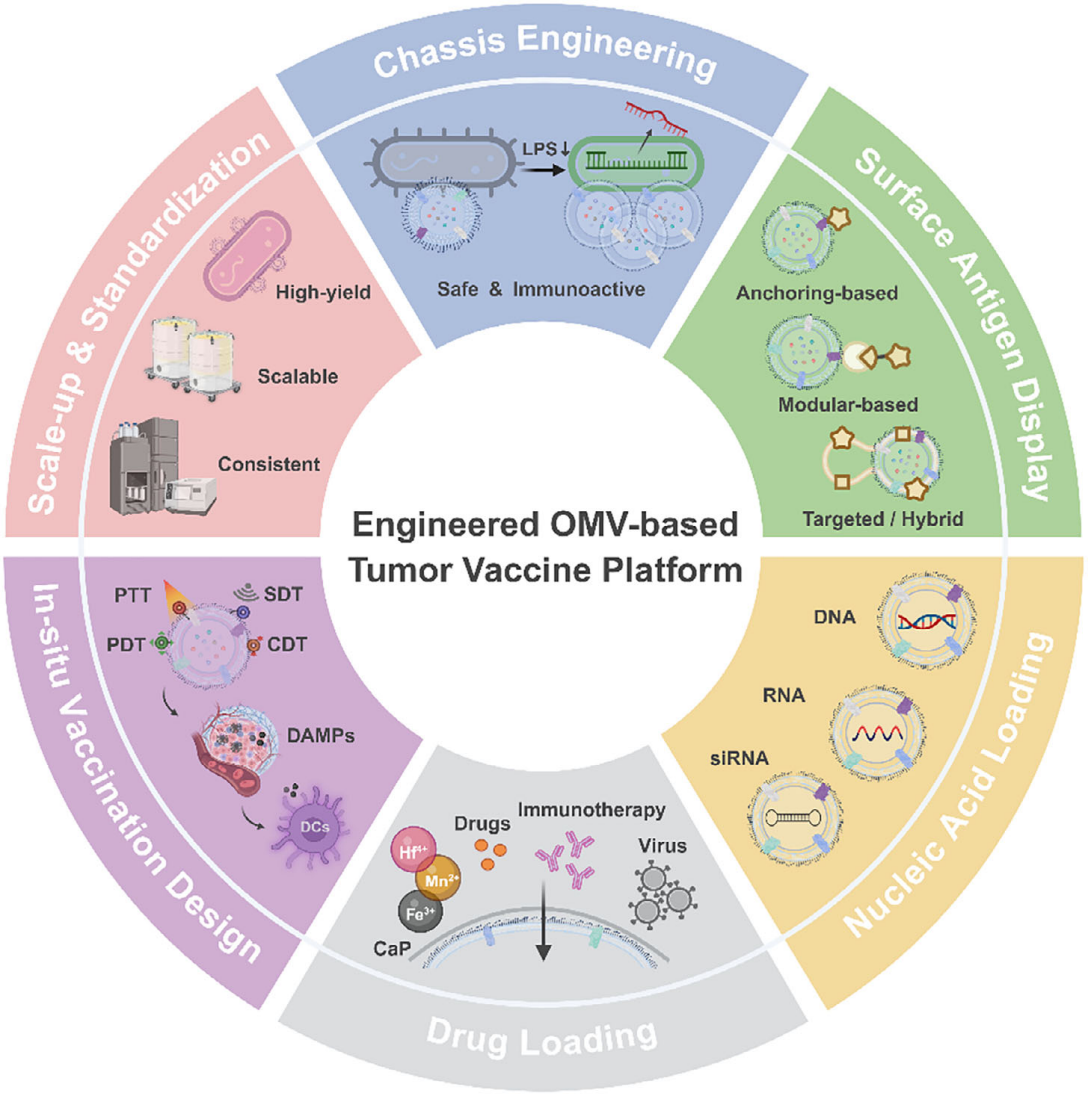
Overall framework of engineering strategies for OMV‐based tumor vaccines. This schematic illustrates a systematic roadmap for transforming OMVs from natural immunostimulatory vesicles into programmable tumor vaccines. Chassis engineering balances safety and immunogenicity while ensuring stable vesicle formation. Surface antigen‐display techniques enable precise and modular antigen presentation. Nucleic acid–loading strategies endow OMVs with genetic and immunoregulatory functions. Drug‐loading modules incorporate metal ions, immune adjuvants, and therapeutic agents to enhance immune responses and support immune memory formation. In situ vaccine designs employ photo‐, sono‐, or chemodynamic modules to trigger ICD, facilitating antigen release and local immune activation. Finally, scalable and standardized manufacturing supports reproducibility and translational readiness, providing a solid foundation for clinical application.

Currently, OMV engineering strategies have evolved into an integrated, hierarchical platform that encompasses genetic chassis design, surface antigen presentation, nucleic acid and drug loading, in situ vaccine development, and scalable, standardized manufacturing. In this system, chassis modifications strike a balance between safety and immunogenicity; surface display enables precise, programmable antigen presentation; nucleic acid and drug loading enhance and expand immune modulation; in situ vaccine techniques increase local tumor immunogenicity; and standardized manufacturing ensures reliable translation. Overall, Figure 3 illustrates the systematic evolution of OMVs from natural immunostimulatory vesicles into programmable immune nanoplatforms, providing both conceptual and technological foundations for the engineering strategies discussed in the following sections.

### 4.2 Surface Display Technology

Surface display technology plays a key role in turning OMVs from “natural immunostimulatory entities” into “programmable vaccine platforms.” By applying rational molecular engineering to the outer membrane structure, anchoring proteins, and antigen presentation sites, this approach enables precise placement and controlled display of antigens on OMV surfaces, offering customizable and adjustable immunopresentation capabilities. The development of this technology can be broken down into four main stages.

#### 4.2.1 Construction of Safe and Presentable Antigen Carriers

The main goal at this phase is to develop a structurally stable and immunologically safe system for surface antigen presentation. Early research employed outer membrane proteins like ClyA, FhuD2, and Trx as anchoring components to reliably display tumor antigens on OMV surfaces, thereby eliciting Th1/CTL‐dominant cellular immune responses [73, 74, 75]. Building on this foundation, strains with low‐endotoxin chassis, such as ClearColi, were used to significantly reduce LPS toxicity without compromising immune activity [76]. Additionally, Chen et al. introduced a polyarginine‐based electrostatic adsorption technique for quickly loading short peptide antigens, which enhanced DCs uptake and cross‐presentation efficiency [77]. These developments collectively established the structural and safety foundation necessary for consistent antigen display and the design of immune‐compatible OMV vaccines.

#### 4.2.2 Realization of Programmable and Modular Antigen Loading

**FIGURE 4:**
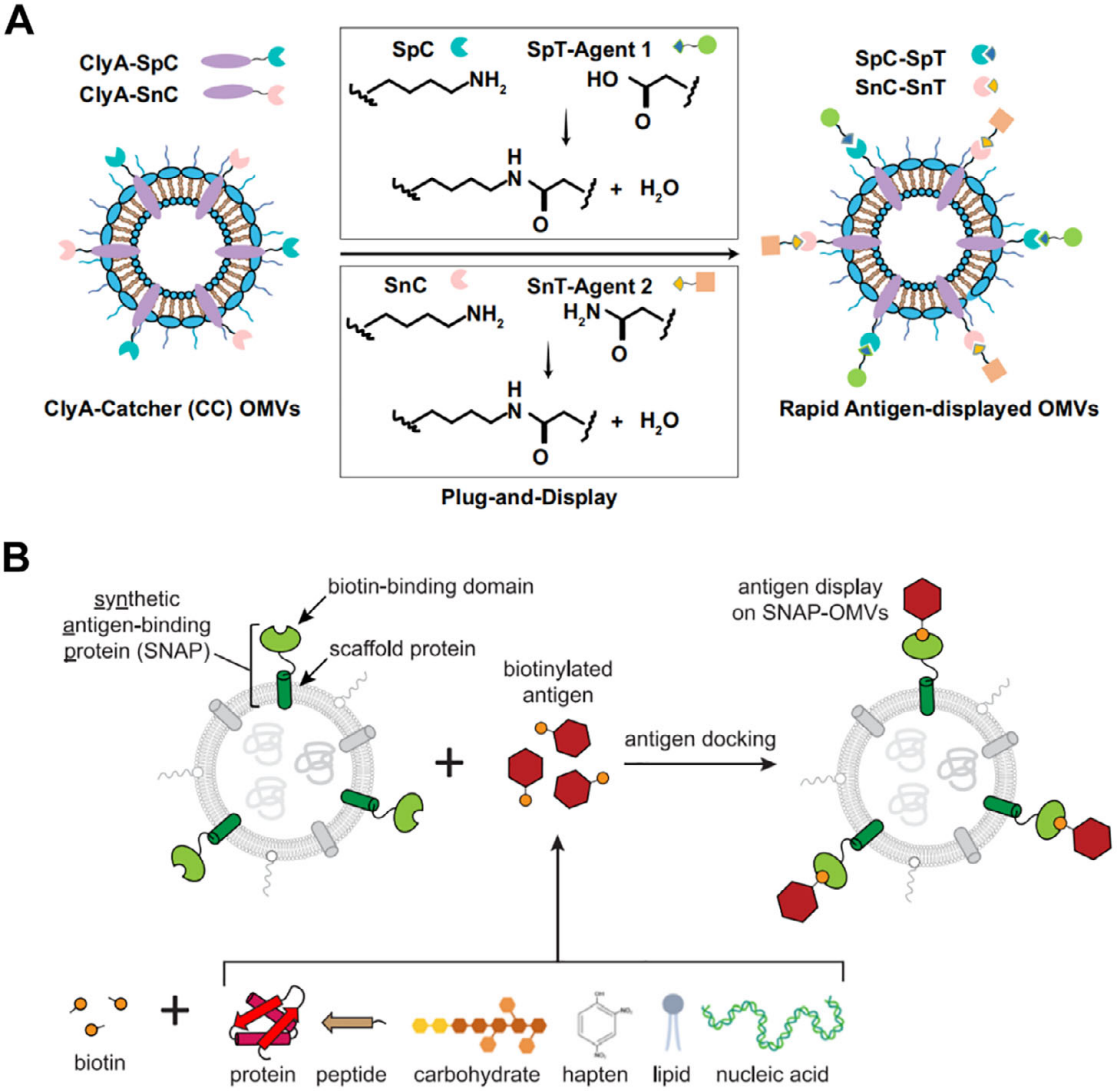
(A) Schematic of modular antigen presentation using the SpyTag/SnoopTag‐Catcher system. The ClyA‐Catcher (CC) OMV platform enables rapid and stable covalent antigen conjugation, allowing programmable and efficient antigen loading. (B) Diagram of the AvidVax (SNAP‐OMVs) system, which uses biotinylated antigens and SNAP receptors via biotin–avidin interactions to enable versatile surface modification of biomolecules and programmable antigen display. Adapted under the terms of the Creative Commons CC BY license [12, 78]. Copyright 2021 and 2023, The Authors.

With advances in chassis construction and anchoring strategies, research has gradually shifted toward modular and controllable designs. The Plug‐and‐Display platform, based on the SpyTag/SpyCatcher covalent coupling system, not only significantly enhanced CD8^+^ T‐cell activation and cross‐presentation but also endowed OMVs with “plug‐and‐play” modularity, enabling flexible antigen exchange and functional customization [12]. Later, the SNAP‐OMV platform enabled rapid antigen swapping without genetic reconstruction, offering a more efficient approach for vaccine development (Figure 4) [78]. Furthermore, the OMVax system enabled regulation of surface charge, providing precise control over antigen adsorption capacity and release kinetics [79]. These technological advances collectively moved OMVs from manually assembled vaccine constructs to standardized, scalable immunomodular systems, laying the foundation for the next generation of programmable tumor vaccines.

#### 4.2.3 Construction of Targeted and Sustained Antigen‐Presentation Systems

During the exploration of OMV‐based vaccine platforms, researchers gradually realized that, in addition to ensuring adequate antigen exposure on OMV surfaces, achieving sustained and efficient immune stimulation is equally crucial for building high‐performance vaccines. To this end, tumor cell membrane–OMV hybrid vesicles were designed, enabling the co‐presentation of personalized tumor antigens and bacterial PAMP signals within a single system, thereby simultaneously activating both innate and adaptive immune responses [80]. Additionally, biodegradable hydrogel encapsulation of such hybrid OMVs was developed to create a sustained‐release system that effectively extended immune stimulation and helped establish durable immune memory [81].

Beyond conventional molecular anchoring–based surface display strategies, these hybrid vesicle designs also reflect an emerging membrane fusion–enabled surface engineering paradigm. In this framework, OMVs function not only as antigen carriers but also as active membrane components capable of interacting with or partially integrating into mammalian cell membranes, forming hybrid membrane interfaces that co‐present tumor‐derived antigens together with bacterial PAMPs. Such membrane‐level integration conceptually links classical surface display technologies with more advanced mixed membrane systems and highlights membrane fusion as a promising direction for expanding OMV vaccine design, despite the absence of fully established multi‐membrane construction frameworks in the OMV field.

Regarding targeting specificity, an αDEC205 receptor–mediated DC‐targeting strategy was used, which supported direct antigen uptake and improved cross‐presentation efficiency [24]. At the same time, ClyA‐fused OMVs engineered with single‐chain antibodies against epidermal growth factor receptor (EGFR) achieved specific recognition of EGFR^+^ tumor cells, thus combining antigen presentation and localized immune activation within a single carrier [82]. Overall, these studies transformed OMVs from basic antigen carriers into smart vaccine units with targeted navigation and prolonged immune‐activation abilities.

#### 4.2.4 Integration of Immunoregulatory Signals and Multifunctional Synergistic Amplification

With the advancement of surface display technologies, research focus has gradually shifted from simple antigen exposure to immune regulation. OMVs are now seen not just as antigen carriers but as dynamic immunoregulatory platforms capable of controlling both local and systemic immune responses. The main challenge is to achieve simultaneous antigen presentation, relief of TME immunosuppression, and local immune activation, thereby enabling a coordinated shift from local activation to systemic immune remodeling.

**FIGURE 5:**
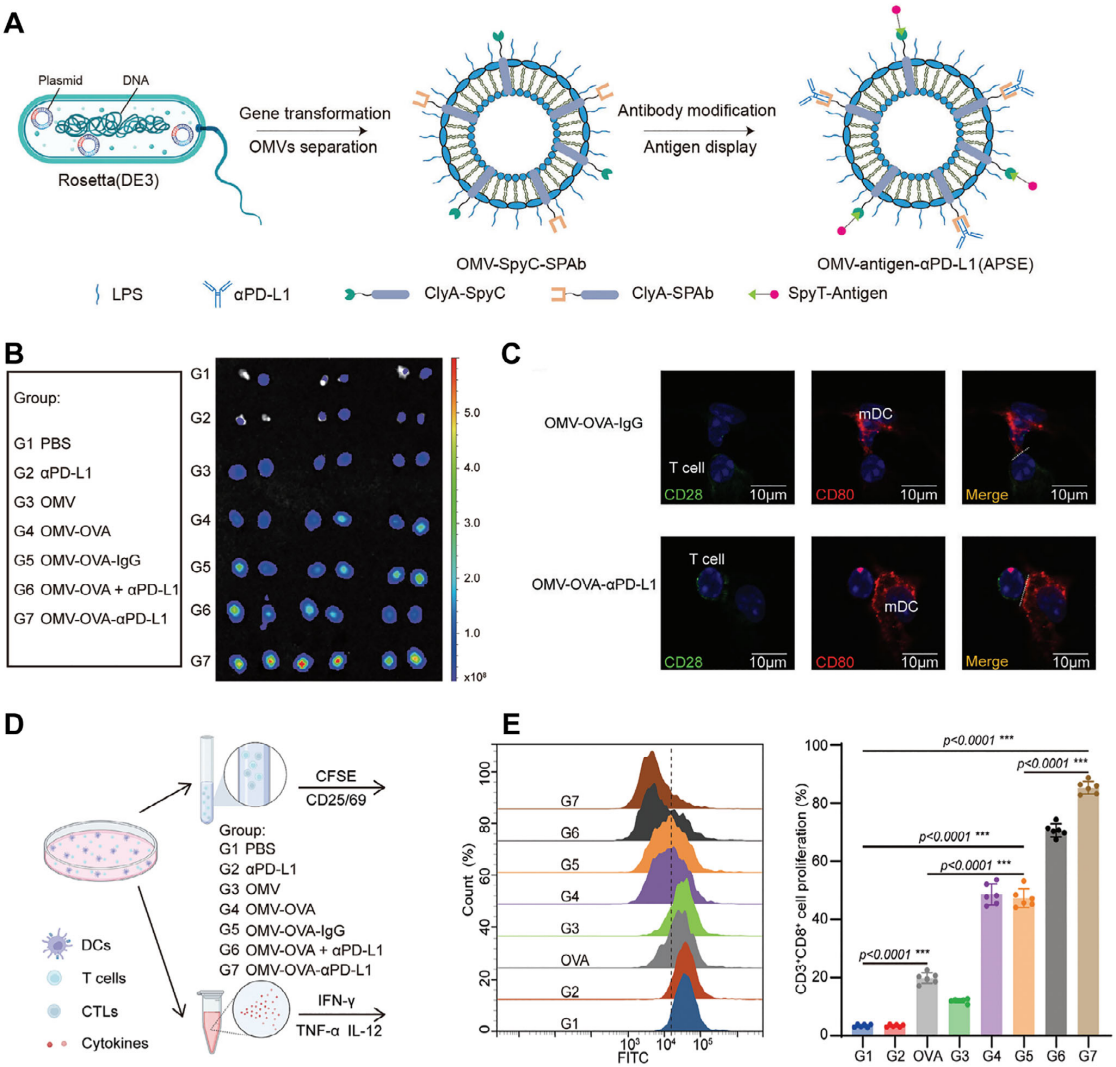
(A) Schematic illustration of the engineered composite OMV vaccine OMV–OVA–αPD‐L1 (denoted as APSE), in which tumor antigens and αPD‐L1 antibodies are co‐displayed on OMVs, thereby integrating antigen presentation and immune checkpoint blockade within a single nanoplatform. (B) Ex vivo fluorescence imaging of draining lymph nodes demonstrates stronger signal accumulation for APSE, indicating improved lymph node delivery and retention compared with control formulations. (C) Immunofluorescence analysis reveals enhanced colocalization between CD80^+^ DCs and CD28^+^ CD8^+^ T cells following APSE treatment, suggesting enhanced antigen presentation‐associated cellular interactions. (D) Schematic of the DC–T cell co‐culture strategy used to evaluate antigen presentation–mediated T cell activation. (E) Flow cytometric analysis of 5,6‐carboxyfluorescein diacetate succinimidyl ester–labeled CD3^+^ CD8^+^ T cells shows that APSE induces the strongest T cell proliferation after 72 h of co‐culture, as indicated by a pronounced leftward shift of fluorescence intensity. Adapted with permission [83]. Copyright 2025, Wiley‐VCH GmbH.

At the local immunoregulatory level, composite OMV‐based nanovaccine systems have been developed using the SpyCatcher/SpyTag plug‐and‐play system. By covalently attaching antigens (OVA) and immune checkpoint antibodies (αPD‐L1) to the same OMV surface, these hybrid vaccines ensure proper spatial coordination between antigen presentation and immune blockade. The resulting vaccine (OMV–OVA–αPD‐L1, APSE) enhances antigen presentation–mediated DC activation and promotes robust T‐cell proliferation, reflecting coordinated immune activation at the local immunoregulatory level (Figure 5) [83]. Consistent with these mechanistic findings, the original study further demonstrated that APSE suppresses tumor growth and lung metastasis and induces durable antitumor immune memory in vivo. This “presentation + regulation” concept was further refined with a LyP‐1–Traptavidin modular platform that induces lysosomal degradation of PD‐L1, directly reducing immune suppression at the cellular level [60]. At the systemic immune‐boosting level, dual‐pathway strategies using PD‐L1nb and CD47nb nanobodies simultaneously block the “immune‐checkpoint–phagocytosis‐inhibition” axes, greatly enhancing CD8^+^ T‐cell effector functions and memory responses [19, 84]. Additionally, aptamer‐modified OMVs were developed to target tumor sites and trigger pyroptosis, releasing DAMPs that boost DC activation and create a positive feedback loop between innate and adaptive immunity [62].

Notably, recent progress has expanded OMV‐based vaccine applications beyond traditional tumor epitopes by exploring unconventional antigens to induce broader immune responses. For example, OMVs displaying full‐length basic fibroblast growth factor (bFGF) antigens simultaneously inhibited cancer‐associated fibroblasts and angiogenesis, remodeling the tumor stroma and promoting immune‐cell infiltration [85]. Another study utilized a GMMA–Tn/STn glycoantigen system, demonstrating the feasibility of OMVs for glycan‐based vaccine development that effectively elicited both humoral and cellular immune responses [86]. Together, these advancements have transformed OMVs from passive antigen carriers into intelligent, self‐amplifying vaccine platforms capable of integrating multiple signaling pathways and orchestrating comprehensive immune modulation.

### 4.3 Nucleic Acid–Loading Strategies

OMVs can serve not only as surface antigen display platforms but also as carriers for nucleic acids, capable of encapsulating DNA or mRNA to enable in vivo antigen expression and sustained presentation. This approach effectively extends immune stimulation and offers better spatiotemporal control of immune responses, transforming OMVs from simple protein antigen carriers into multifunctional systems capable of triggering immune activation at the genetic level.

In the antigen‐expression design, the LOMV@PD‐1 system delivers plasmid DNA encoding PD‐1, enabling tumor cells to produce PD‐1 endogenously. This mechanism achieves a self‐blocking effect on PD‐L1 and enhances the activation of CTLs and NK cells [87]. Additionally, an mRNA surface‐display system was developed using the RNA‐binding protein L7Ae and the lysosomal escape protein LLO, enabling mRNA to be efficiently translated within DCs and induce cross‐presentation, thereby significantly boosting CD8^+^ T‐cell responses [88].

**FIGURE 6:**
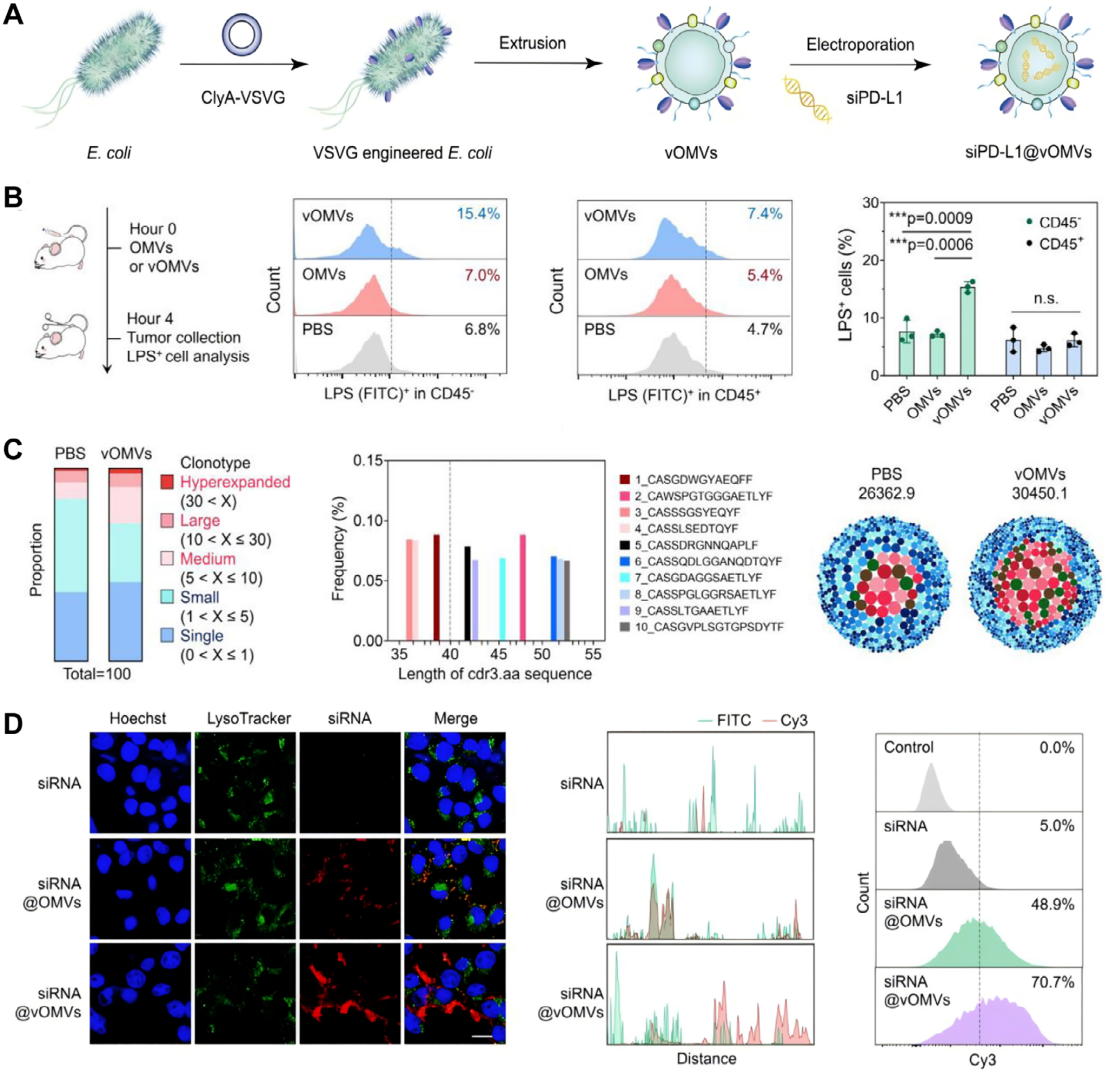
(A) Schematic illustration of the VSVG‐engineered outer membrane vesicle (vOMV) platform for siPD‐L1 delivery, in which ClyA–VSVG fusion confers acid‐responsive membrane fusion and facilitates cytosolic cargo release. (B) Flow cytometric analysis shows enhanced deposition of LPS within CD45^−^ tumor cells, but not CD45^+^ immune cells, following vOMV treatment, indicating selective tumor foreignization. (C) Analysis of T cell receptor repertoires in tumor‐draining lymph nodes reveals broadened clonal expansion and increased TCR diversity after vOMV administration. (D) Confocal imaging and flow cytometry demonstrate that vOMVs markedly enhance cytosolic delivery of siRNA cargo by reducing endosomal/lysosomal sequestration compared with conventional OMVs. Adapted under the terms of the Creative Commons CC BY license [89]. Copyright 2025, The Authors.

In recent years, nucleic acid–loading strategies have advanced, improving the regulation of tumor immunosignaling. For example, vesicular stomatitis virus glycoprotein (VSVG)–modified OMVs (vOMVs) exploit an acid‐sensitive membrane fusion mechanism to deliver siPD‐L1 directly into the cytoplasm of tumor cells, thereby avoiding endosomal trapping and enabling efficient PD‐L1 gene silencing. This strategy was shown to induce tumor foreignization, reshape T cell receptor repertoires, and enhance cytosolic nucleic acid delivery (Figure 6). In the original study, this system was further reported to suppress tumor growth and metastasis in CT26 and 4T1 models, accompanied by increased CD8^+^ T‐cell infiltration and IFN‐γ production [89]. Additionally, stimuli‐responsive OMVs, such as photothermal or pH‐sensitive OMVs, can coordinate the co‐delivery of siRNA and chemotherapeutic drugs. For example, co‐encapsulating siRedd1 and paclitaxel allows a sequential “chemotherapy–immunotherapy” release mode: first inducing ICD, then reprogramming TAM metabolism to promote M1 polarization and reduce metastasis [90, 91]. Overall, nucleic acid–loading strategies have transformed OMVs from traditional antigen‐presenting platforms into multi‐functional vaccine systems that combine gene regulation with immune activation. This approach opens new possibilities for creating sustained, self‐regulating antitumor vaccines with programmable immunomodulatory potential.

### 4.4 Drug‐Loading Strategies for Immunomodulation

To further enhance the immunotherapeutic potential of OMV‐based tumor vaccines, recent strategies have advanced beyond simple cargo delivery to include comprehensive immune orchestration. Functionalized OMVs are now designed not only to deliver therapeutic molecules but also to regulate antigen release, increase immune activation thresholds, boost signaling pathways, and support memory formation. In this context, OMVs serve as “vaccine converters” and “immune amplifiers” within the TME.

#### 4.4.1 Signal Activation: Igniting the Initial Immune Response

In the TME, OMVs loaded with manganese (Mn) release Mn^2^^+^ under acidic or hypoxic conditions, triggering mitochondrial or cytosolic DNA leakage and activating the cyclic GMP–AMP synthase (cGAS)–stimulator of interferon genes (STING) pathway. This results in the production of type I interferons, DC maturation, and improved antigen cross‐presentation, collectively enhancing CD8^+^ T‐cell immunity [92, 93, 94]. Similarly, CaP–melanin–mineralized OMVs boost tumor immunogenicity and reduce toxicity during photothermal therapy (PTT) [95]. Building on this idea, metal–phenolic OMVs (Hf–OMVs), constructed through coordination between tannic acid and hafnium ions, effectively alleviate tumor hypoxia by catalyzing the decomposition of H_2_O_2_ to generate O_2_ and thereby enhance radiotherapy‐induced ICD. By increasing tumor‐associated antigen release and promoting dendritic cell maturation, Hf–OMVs rejuvenate the DC–T cell axis and elicit robust systemic antitumor immune responses [64].

#### 4.4.2 Signal Amplification: Extending Local Activation Into Systemic Immunity

Local immune activation alone rarely achieves lasting systemic protection. Therefore, advanced designs integrate OMVs with oncolytic viruses, autophagy pathways, or pyroptosis–apoptosis–necroptosis (PANoptosis) signaling to trigger widespread antigen release and immune boosting. For example, OMVs@P2O‐Ads combined with adenoviral therapy enhances autophagy and cross‐presentation [96], while Fn‐OMV–HSV hybrid systems induce PANoptosis, boost interferon signaling, and strengthen T‐cell activation [97]. Synthetic OMVs carrying STING agonists further increase IFN‐I production, significantly improving DC–T‐cell communication and systemic immunity [98]. These multi‐step amplifiers transform local immune activation into a coordinated, whole‐body antitumor response.

#### 4.4.3 Integration and Memory Formation: Reversing Suppression and Consolidating Immunity

The immunosuppressive nature of the TME—characterized by increased PD‐L1 expression, M2‐type TAM polarization, and lactate buildup—often diminishes vaccine effectiveness. To address these challenges, engineered OMVs have been combined with checkpoint blockade or PD‐L1 degradation modules (e.g., OMV‐LYTAC) and metabolic/epigenetic regulators, successfully reducing suppression and boosting immune activation [59, 61, 99].

**FIGURE 7:**
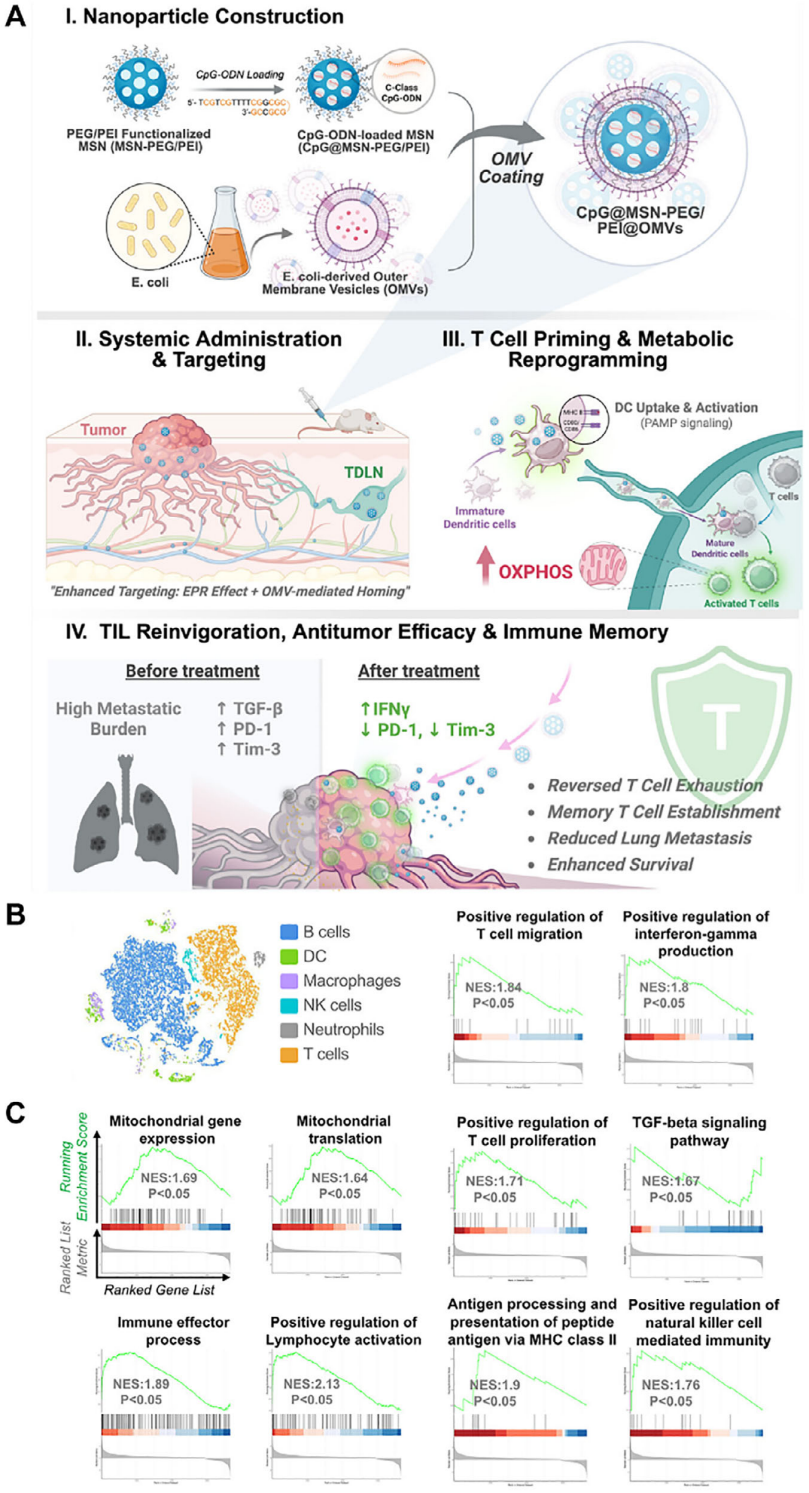
(A) Schematic illustration of the platform design and underlying mechanism. CpG oligodeoxynucleotides (TLR9 agonists) are loaded into an MSN‐PEG/PEI core and cloaked with OMVs to create a dual‐pathway immune‐activating nanoplatform. This design promotes DC maturation and T‐cell activation. (B) t‐SNE analysis of scRNA‐seq data showing distinct immune cell populations in tumor‐draining lymph nodes (TDLNs). (C) GSEA enrichment results from integrated single‐cell transcriptomic analysis revealed significant enrichment of mitochondrial and immune activation pathways within the T‐cell cluster after CpG@MSN‐PEG/PEI@OMVs treatment, including OXPHOS, energy metabolism, and immune response–related pathways (NES > 1.5). These findings indicate that the platform restores T‐cell function and establishes durable antitumor immunity through metabolic–immune dual reprogramming. Adapted under the terms of the Creative Commons CC‐BY 4.0 license [100]. Copyright 2025, The Authors.

Furthermore, vaccines related to trained immunity that utilize OMVs and GM‐CSF can reprogram bone marrow progenitors and monocytes through metabolic and epigenetic remodeling, boosting phagocytic capacity and systemic antitumor immunity [13]. A CpG@MSN‐PEG/PEI@OMVs cloak platform combining CpG (a TLR9 agonist) with OMVs achieved dual‐pathway immune synergy. Single‐cell transcriptomics showed significant upregulation of T‐cell oxidative phosphorylation (OXPHOS) and immune‐activation genes (NES > 1.5), along with suppression of NR4A exhaustion factors, thereby restoring effector and memory T‐cell functions. This dual reprogramming of metabolism and immunity offers a mechanistic basis for lasting immune memory. Simultaneously, OMVs loaded with CpG or other TLR agonists directly encourage DC maturation and cross‐presentation, expanding the memory T‐cell pool and extending immune response durability (Figure 7) [100]. Other innovations—such as photodynamic therapy–based OMVs (PDT‐OMVs) for ROS generation, G/COMVs delivered via microneedle arrays, and tegafur‐ or aptamer‐functionalized OMVs—optimize antigen release, tissue penetration, and ICD induction, collectively supporting long‐term immune memory [62, 101, 102, 103].

In summary, by integrating energy‐conversion modules (PTT, PDT, radiotherapy), signal‐amplification modules (viral, autophagic, PANoptosis, STING activation), and suppression‐relief plus metabolic/epigenetic‐regulation modules, researchers are transforming traditional drug carriers into multidimensional immunovaccine platforms. As advances continue in ICD induction and antigen release–capture dynamics, these systems are gradually evolving into in situ vaccine architectures, laying a foundation for durable immune memory and ongoing tumor protection.

### 4.5 In Situ Vaccine Strategy

The in situ vaccine strategy aims to convert the tumor itself into a “vaccine factory” by inducing ICD and releasing tumor‐associated antigens and DAMPs—including CRT exposure, HMGB1 release, ATP secretion, and DNA leakage—which then activate DCs and IFN‐I responses, leading to CD8^+^ T‐cell activation and long‐term immune memory [104, 105]. Unlike the drug‐loading strategies described in Section 4.4, OMV‐based in situ vaccines focus on spontaneous antigen release and capture within the TME, thereby establishing a sustained, antigenically diverse immune network.

**FIGURE 8:**
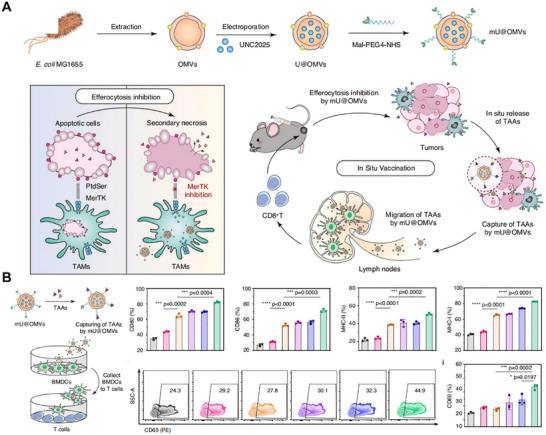
(A) Schematic diagram of the mU@OMV system, where the MerTK inhibitor UNC2025 is encapsulated into *E. coli* MG1655–derived OMVs and then modified with Mal‐PEG_4_‐NHS to create maleimide‐functionalized OMVs (mU@OMVs). This system blocks MerTK‐mediated efferocytosis of apoptotic cells, causes secondary necrosis, and promotes the release of tumor‐associated antigens (TAAs). The maleimide groups on the OMV surface enable in situ covalent antigen capture via thiol–maleimide linkage, boosting antigen retention at the tumor site and facilitating its transport to draining lymph nodes. (B) In vitro validation of the in situ vaccine function. TAAs captured by mU@OMVs were co‐cultured with bone marrow–derived dendritic cells (BMDCs), resulting in significant increases in CD80, CD86, MHC‐I, and MHC‐II expression. In the BMDC/T‐cell co‐culture system, CD8^+^ T‐cell activation (indicated by increased CD69^+^ proportion) was notably enhanced, demonstrating that combining efferocytosis blockade with antigen capture effectively rebuilds, in vitro, the in situ loops of antigen presentation and immune activation. Adapted under the terms of the Creative Commons CC BY license [108]. Copyright 2023, The Authors.

Antigen release is crucial for activating immune responses. The PTT‐OMV system triggers ICD under near‐infrared irradiation, promoting CRT exposure and HMGB1 release, which dramatically enhances DC maturation and antigen presentation efficiency [106]. Similarly, the PDT‐OMV system relies on photosensitizer‐mediated ROS production and ATP secretion to improve cross‐presentation [107]. Additionally, metal‐catalytic OMV platforms (CDT) or pyroptosis‐inducing OMV platforms can increase DAMP expression, boost innate immune signaling, and strengthen T‐cell activation [63, 65]. Next, the capture and stabilization of antigens determine how long immune responses last. By adding maleimide functional groups, OMVs can covalently attach antigens at tumor sites, significantly extending antigen retention and enhancing presentation efficiency, which promotes DC maturation and CD8^+^ T‐cell expansion to create a self‐sustaining antigen–presentation–activation loop (Figure 8) [108]. Furthermore, mineralized or metal–polyphenol cage structures reduce endotoxin‐related toxicity and optimize antigen release kinetics, leading to more stable and effective antigen presentation [65].

Signal amplification is essential for maintaining immune strength. Intrinsic PAMPs on OMVs can directly activate the TLR–IFN‐I axis and promote DC maturation [109]. The integration of sonodynamic or chemodynamic modules—exemplified by Pt–porphyrin–mediated ROS generation—can enhance the cGAS–STING signaling cascade and trigger pyroptosis along with other inflammatory cell death programs, resulting in continuous immune amplification and improved antigen presentation within the TME [63]. Notably, this study used extracellular vesicles from *S. aureus*, a Gram‐positive bacterium lacking an outer membrane and LPS layer but with immunostimulatory mechanisms similar to those of OMVs, thereby extending OMV‐based strategies to Gram‐positive bacterial systems. ^63^Furthermore, a CRISPR–OMV self‐assembly platform has recently been developed to deliver CXCL9 and IL‐12 directly within tumors while silencing immunosuppressive genes, thereby boosting T‐cell recruitment and effector functions and promoting cascade immune amplification [110]. Ultimately, releasing immune suppression and maintaining immune memory are crucial for long‐lasting effectiveness. Several studies have shown that OMVs combined with PD‐L1 blockade or degradation modules, TAM reprogramming, and hydrogel‐based sustained release systems achieve extended T‐cell persistence and significantly reduce tumor recurrence in rechallenge model [66, 105, 110, 111].

In summary, OMV‐based in situ vaccines leverage tumors as natural sources of antigens, eliminating the need for exogenous antigens and offering distinct benefits for addressing antigenic diversity and maintaining immune responses. Although additional optimization is necessary to improve safety and facilitate clinical translation, this approach shows significant potential as a link between tumor immunotherapy and vaccine development, presenting a new direction for next‐generation cancer vaccines.

### 4.6 Strategies to Enhance the Yield and Consistency of OMVs

Achieving high yield and batch‐to‐batch consistency is crucial for the clinical translation of OMVs. Besides scalable production and controllable purity, OMV preparation must preserve immune activity and structural stability. In recent years, strategies to increase OMV yield have shifted from isolated genetic or process optimizations to a more integrated approach that combines outer membrane engineering, environmental stress regulation, and standardized quality control. By systematically modifying bacterial membrane architecture and vesiculation behavior, researchers have established a strong technological foundation for the efficient and predictable production of OMVs suitable for vaccine manufacturing.

#### 4.6.1 Genetic and Membrane Structural Regulation

The mechanical connection between the outer membrane and the peptidoglycan (PG) layer is a key factor in controlling vesicle release efficiency. Weakening this connection lowers envelope tension, increases membrane curvature, and promotes spontaneous OMV release. Key structural links include Lpp, OmpA, NlpI, and the Tol–Pal complex, which work together to maintain outer membrane–PG integrity: Lpp forms covalent bonds with PG; the C‐terminal domain of OmpA binds noncovalently to diaminopimelate residues in PG; NlpI regulates PG metabolism; and the Tol–Pal system provides mechanical support across the envelope. Deleting these genes greatly increases vesiculation—Δ*lpp* boosts OMV production by about 10–40 times [112, 113]; Δ*ompA*, 10–30 times [114]; Δ*nlpI*, 7–100 times [115]; and Δ*tolA*/*tolR*/*pal*, 14–33 times [116, 117, 118]. These results demonstrate that weakening OM–PG connections enables structurally driven vesiculation via mechanical relaxation.

Disruption of outer membrane lipid asymmetry significantly promotes OMV release. The Mla system (e.g., *mlaA*, *mlaE*) maintains the phospholipid‐LPS distribution between outer membrane leaflets. Its deletion leads to phospholipid accumulation in the outer leaflet, resulting in curvature stress and vesicle formation. In *E. coli*, a single *mlaE* mutation increases OMV production by about 8 times, while a double deletion (Δ*mlaE*Δ*nlpI*) has a synergistic effect, boosting vesicle output by 14 to 30 times [115, 119, 120]. When lipid imbalance and outer membrane–PG detachment coincide, envelope homeostasis fails, triggering a “pressure‐relief vesiculation” process—an integrated genetic mechanism linking mechanical relaxation and lipid asymmetry. Additionally, the regulation of envelope stress response systems influences vesiculation. In *E. coli*, removal of the DLP12 lysis module (holin–endolysin–spanin system) increased OMV production approximately fivefold under steady‐state conditions [121]. This suggests that controlled mechanical weakening and stress release can serve as additional methods to enhance OMV formation.

Together, weakening envelope constraints through structural relaxation, lipid imbalance, and stress regulation enables high‐efficiency, controllable OMV release without reducing immunogenicity. These molecular and process‐level improvements lay the foundation for scalable, high‐quality OMV vaccine production.

#### 4.6.2 Environmental Stimulation and Culture Optimization

Optimizing culture conditions and process parameters is a crucial approach to increasing OMV yield. Environmental factors influence vesiculation by altering the redox state and lipid composition of the outer membrane. Studies indicate that oxygen levels and iron availability regulate oxidative balance—moderate hyperoxia or iron deprivation trigger stress responses that enhance OMV release [122, 123, 124, 125]. Furthermore, antibiotic‐induced stress, via membrane disruption, SOS activation, and inhibition of cell‐wall synthesis, promotes vesicle secretion [126, 127]. Recent research has shown that chemical regulation of peptidoglycan remodeling using functional inhibitors allows reversible control of OMV production, increasing yield up to 60‐fold across multiple strains [128]. This method provides pharmacological reversibility and broad strain applicability without genetic modification, while also enabling co‐loading of photosensitizers or immunostimulatory molecules—balancing production efficiency with functional versatility.

The introduction of bioreactor‐based continuous culture has further enhanced OMV yield and batch consistency. Compared to conventional batch systems, fed‐batch or continuous processes maintain long‐term steady states by precisely regulating temperature, pH, and dissolved oxygen. In Neisseria meningitidis models, continuous culture enabled stable operation for over 600 h, tripled volumetric productivity, and maintained consistent vesicle size and protein profiles [129]. Moreover, high dissolved oxygen induction combined with tangential flow filtration (TFF), DNase treatment, and gel chromatography increased OMV recovery to 90%, while reducing contaminants like DNA and membrane debris [123, 130]. These advances create a solid foundation for industrial‐scale, standardized, and reproducible OMV vaccine production.

#### 4.6.3 Quality Control and Standardization Systems

The clinical translation of OMV‐based vaccines requires robust and standardized quality control systems to ensure batch‐to‐batch consistency and immunological reliability. Given the intrinsic compositional complexity of OMVs, quality control assessment generally relies on a combination of physicochemical, safety‐related, and composition‐based parameters rather than a single metric. Standard physicochemical characterization includes vesicle size distribution, morphology, and homogeneity, typically evaluated by dynamic light scattering and transmission electron microscopy (TEM), together with zeta potential (ζ potential) as an auxiliary indicator of surface charge and colloidal stability. In parallel, LPS content and endotoxin activity remain critical safety parameters and are commonly assessed using biochemical quantification and TLR activation assays to balance immunogenicity and tolerability.

Beyond these general metrics, recent studies have highlighted the value of outer membrane proteomic fingerprints for OMV identity verification and consistency evaluation. Standardization frameworks have proposed integrating quantitative proteomic profiles with physicochemical parameters to improve batch traceability and immunological predictability [131]. Proteomic analyses further demonstrate that OMVs exhibit strain‐ and species‐specific enrichment patterns of high‐abundance outer membrane–associated proteins. For example, OMVs derived from Escherichia coli are consistently enriched in structural outer membrane proteins such as OmpA and OmpC/F, whereas OMVs from Fusobacterium nucleatum display prominent enrichment of outer membrane–associated and self‐transport proteins, reflecting species‐dependent vesiculation mechanisms and membrane composition [132]. These findings support the concept of defining platform‐specific signature protein panels, rather than universal markers, as a rational strategy for OMV quality control.

Overall, OMV manufacturing is transitioning from passive vesicle release toward more controllable biomanufacturing processes through membrane engineering, culture optimization, and process standardization. Nevertheless, variations in bacterial strains, growth conditions, and purification procedures may still introduce structural and immunological heterogeneity. During the translation of OMV‐based vaccines toward GMP‐grade production, achieving highly reproducible molecular composition while preserving immunogenic function remains a central challenge for standardization and regulatory evaluation.

## 5 Challenges and Future Perspectives

### 5.1 Challenges in Addressing Heterogeneity and Ensuring Quality Consistency

The complex biological origin of OMVs makes their composition highly sensitive to bacterial strain background and manufacturing conditions, leading to significant heterogeneity. To address this, current research has shifted from focusing on physical traits such as particle size and protein ratios to developing function‐based quality assessment systems rooted in immunological performance. For example, TLR activation profiling and immune fingerprinting are now used to measure the immunological consistency of OMVs, providing a more accurate reflection of their biological stability. During large‐scale production, quality control has shifted from passive detection to proactive design—integrating quality assurance directly into the manufacturing process through mechanistic understanding rather than relying solely on endpoint testing. Rather than screening for high‐quality vesicles after production, the modern approach aims to engineer processes that inherently produce high‐quality OMVs. By understanding vesicle formation kinetics, identifying critical process parameters, and creating predictive process models, the entire workflow—from vesicle creation to purification—can be monitored in real time. Additionally, computational modeling and data‐driven analytics enable continuous monitoring and predictive control of OMV production, dynamically adjusting process parameters and shifting quality control from final inspection to real‐time process management. This paradigm shift signals that OMV manufacturing is moving from empirical optimization to a mechanistic and intelligent biomanufacturing approach, paving the way for reproducible, scalable, and clinically compliant OMV‐based vaccines.

Notably, the translational feasibility of OMV platforms has been demonstrated beyond conceptual and preclinical studies through multiple licensed or late‐stage OMV‐based vaccines developed against serogroup B *Neisseria meningitidis*, including VA‐MENGOC‐BC, MenBvac, MeNZB, and the currently approved Bexsero [133, 134, 135]. These vaccines have been implemented in national immunization programs or large‐scale clinical studies across different regions, collectively demonstrating that OMV formulations can achieve acceptable long‐term safety profiles, robust immunogenicity, and scalable, standardized manufacturing.

Although originally developed for the prevention of infectious diseases, the evolutionary trajectory of these vaccines—from outbreak‐driven interventions to products supported by mature manufacturing processes and well‐defined regulatory pathways—provides valuable conceptual and manufacturing‐related translational lessons for the development of OMV‐based cancer vaccines. In particular, these precedents highlight the feasibility of platform standardization and batch‐to‐batch quality consistency, which are critical considerations for clinical translation.

### 5.2 Immunological Interplay Between Endogenous and Exogenous OMVs

OMVs derived from commensal bacteria play vital roles in maintaining host immune homeostasis. For instance, *Bacteroides thetaiotaomicron*–derived OMVs can induce IL‐10 and TGF‐β expression, enhance epithelial barrier integrity, and suppress excessive inflammation; similarly, OMVs from *Parabacteroides goldsteinii* can translocate across the intestinal epithelium and inhibit NF‐κB and STAT3 signaling, thereby reducing psoriasis‐like inflammation [136, 137]. These findings suggest that commensal‐derived OMVs not only activate mucosal immunity but also support systemic anti‐inflammatory balance, acting as key immunological messengers linking local defense and overall homeostasis. In contrast, exogenous OMVs—typically derived from engineered or pathogenic bacteria—are widely used in vaccine development and tumor immunotherapy. Advances in transdermal electroporation microneedles and oral delivery strategies have broadened the application of OMV‐based tumor vaccines, enabling efficient antigen presentation and immune activation through both skin and gut mucosal pathways [103, 138]. Overall, the immune‐regulating functions of endogenous OMVs and the immune‐activating potential of exogenous OMVs are complementary, providing a theoretical basis for creating “homeostasis‐oriented antitumor OMVs” that balance effectiveness with immune tolerance.

Interestingly, some exogenous OMVs can trigger strong immune responses while causing low systemic inflammation. For example, *Neisseria meningitidis*–derived OMV vaccines can provide cross‐protection against *Neisseria gonorrhoeae* under low‐inflammatory conditions [139], indicating possible mechanisms for sharing antigenic information and reusing immune memory between bacterial species. This “low‐inflammatory, high‐specificity, antigen‐sharing” pattern of immune activation offers new insights for developing next‐generation OMV‐based tumor vaccines that balance safety and effectiveness.

However, introducing exogenous OMVs may also disrupt this fragile balance. Some studies suggest that exogenous OMVs, which share structural PAMP motifs with commensal OMVs, might compete for epithelial TLR binding sites or modulate immune thresholds, causing temporary inflammatory shifts—though direct evidence remains limited [140, 141]. Notably, a 2025 *Nature* study showed that host intestinal epithelial cells secrete Apolipoprotein L9a/b (*ApoL9a/b*), which detects Ceramide‐1‐phosphate (*Cer1P*) on the outer membranes of *Bacteroidales* and triggers OMV release. This process activates IFN‐γ signaling and increases MHC‐II expression, helping maintain intestinal immune homeostasis. In *ApoL9a/b*‐deficient mice, inflammation worsened and intraepithelial lymphocytes decreased; notably, adding *Bacteroidales* OMVs restored immune balance and improved outcomes [142]. This study provides the first direct physiological evidence that exogenous OMVs can reestablish immune homeostasis when host regulation is impaired, highlighting the complex and reciprocal relationship between endogenous and exogenous OMVs. In conclusion, deciphering this bidirectional immune regulation will be crucial for the future development of safe, effective, and precisely controllable OMV‐based vaccines and immunotherapies, representing a key frontier at the intersection of tumor immunology and host–microbiota interaction research.

### 5.3 Integration of OMVs With AI: Future Prospects

#### 5.3.1 AI‐Driven Antigen Prediction and Intelligent Design

The convergence of AI and multi‐omics technologies is driving the development of OMV‐based tumor vaccines from experimental research to intelligent and systematic innovation. Current AI models now encompass the entire prediction process—from identifying gene mutations to analyzing antigen structures [143]. For example, the DeepSEA model predicts how DNA mutations influence transcription factor binding and chromatin accessibility, offering insights into the upstream regulation of immune‐related genes [144]; SNAF utilizes RNA‐seq data to identify tumor‐specific antigens produced by abnormal alternative splicing [145]; deepAntigen employs deep graph networks to model the binding interactions among peptides, MHC, and TCR, enabling accurate prediction of personalized immune responses [146]; AlphaFold 3, with its multimodal structural generation, provides further understanding of the spatial stability of peptide–receptor complexes, aiding epitope screening through structural validation. The SYNBIP 2.0 database offers modular binding protein templates to verify the structural feasibility of engineered antigen presentation [147]. Using these computational predictions, the OMV platform can execute a complete closed‐loop workflow from algorithmic screening to functional validation. For instance, recombinant OMVs (rOMVs) derived from ClearColi were engineered with the ClyA system to display computationally predicted neoepitopes in parallel. In murine CT26 tumor models, this approach successfully elicited a strong Th1‐type immune response and resulted in complete tumor regression [148]. This strategy demonstrates the synergistic potential of combining AI‐based prediction with OMV engineering.

However, this integrated framework still faces three main challenges:

(1) Variability in multi‐omics data and the lack of cross‐platform standards limit the model's generalizability and clinical application.

(2) Most AI predictions remain confined to the “sequence–structure” level, lacking interpretability of immune responses and experimental validation; and

(3) The absence of standardized, closed‐loop pipelines between computational prediction and OMV‐based verification hinders reproducibility and traceability.

Future development should aim to create a self‐learning AI–OMV experimental feedback system that enables dynamic cycles of antigen screening, structural modeling, and immune validation using multimodal models. By integrating AlphaFold 3 and DeepSEA for mutation function and spatial conformation prediction, and applying the Quality by Design concept into OMV production processes, AI is anticipated to deliver quality‐focused solutions throughout the entire vaccine development pipeline—from antigen design to GMP‐level manufacturing—providing verifiable and traceable intelligent frameworks for personalized tumor vaccine development.

#### 5.3.2 Intratumoral Microbiota and Shared Antigen Recognition

Multi‐omics studies have shown that tumor tissues harbor long‐term symbiotic microbiota, whose OMV release contributes to remodeling the tumor immune microenvironment. Some microbial proteins share sequence or structural homology with tumor antigens at the epitope level, forming shared antigens that can induce cross‐reactive immune recognition and memory reuse, thus providing new immunological foundations for vaccine development [149].

Recent work from the *MicroEpitope* database has systematically mapped cancer‐associated microbial epitopes. Using immunopeptidomics data from 1,190 samples across 24 cancer types, researchers identified 51,497 bacterial and 767 viral epitopes, mainly from *Bacillus subtilis*, *Buchnera aphidicola*, and human cytomegalovirus. These microbial epitopes can bind to host HLA molecules and be presented in tumor tissues, suggesting that tumor‐associated microbiota may influence host immune responses through antigenic homology or molecular mimicry mechanisms [150]. Another study provided direct experimental evidence for the role of commensal‐derived OMVs in tumor immunomodulation. OMVs from *Bacteroides fragilis* (*BEVs*) enriched in breast cancer tissues promoted DC maturation and CD8^+^ T‐cell activation but were accompanied by systemic inflammation. To address this, researchers designed programmed MnO_2_‐coated OMVs that release Mn^2^^+^ in response to pH changes, activating the cGAS–STING pathway and enhancing antigen presentation. In the MCF‐7 tumor model, this vaccine induced a strong Th1‐type immune response and achieved a 78% tumor inhibition rate when combined with αPD‐L1 therapy, while also significantly suppressing metastasis [151].

Together, these findings suggest that commensal‐derived OMVs can serve not only as carriers of shared antigens but also as engineered boosters of immune responses within the TME. Moving forward, combining AI‐driven antigen‐prediction models with resources such as the *MicroEpitope* database will enable systematic screening of shared antigens and evaluation of their immune affinities, ultimately creating a comprehensive framework—from molecular mechanisms to functional validation—for developing OMV vaccines based on shared antigens.

## 6 Conclusion

OMVs, with their natural ability to stimulate the immune system and activate multiple immune pathways, have become essential enhancers of tumor vaccine effectiveness. Combining self‐adjuvanticity, engineering flexibility, and high biosafety, OMVs can activate both innate and adaptive immunity simultaneously while maintaining immune balance, thus overcoming the traditional trade‐off between safety and efficacy. With ongoing advances in genetic modification, surface display, nucleic acid loading, and immunotherapeutic drug integration, OMVs have evolved from natural immune boosters into customizable immunomodulatory platforms capable of precisely and controllably enhancing the immune response across various tumor types. Additionally, integrating AI‐based antigen prediction, personalized design, and quality control is driving the development of intelligent vaccine systems that optimize immune activation while preserving homeostasis, maximizing effectiveness while ensuring safety. Essentially, OMVs are not just carriers for tumor vaccines but also strategic tools that transform cancer immunotherapy. Continued research into mechanisms and cross‐disciplinary innovation will accelerate the transition of OMVs from experimental stages to clinical application, heralding a new era of precise, controllable, and durable antitumor immunity.

## Funding

This work was supported by grants from the National Natural Science Foundation of China (Grant Nos. 32222045 and 32171384, X. Z.; 32471450 and 82402462, K. C.), the Beijing Natural Science Foundation (Grant No. F252053, X. Z.), the Beijing Municipal Science & Technology Commission (Grant No. Z231100007223011, X. Z.), and the Beijing Natural Science Foundation (Grant No. 7244523, K. C.).

## Conflicts of Interest

The authors declare no conflicts of interest.

## Data Availability

Data sharing does not apply to this article as no new data were created or analyzed in this study.
